# Exploring the mechanism of action of Jinqiancao granules against liver cirrhosis based on the liver-gut axis

**DOI:** 10.3389/fphar.2025.1686535

**Published:** 2025-11-26

**Authors:** Fan-E. Cheng, Yaxi Zhang, Shihao Zheng, Wenying Qi, Xiaomei Zhang, Size Li, Qiuyue Wang, Xiangyun Zou, Xiaoke Li, Yongan Ye, Xiaobin Zao

**Affiliations:** 1 Dongzhimen Hospital, Beijing University of Chinese Medicine, Beijing, China; 2 Beijing University of Chinese Medicine, Beijing, China; 3 Liver Diseases Academy of Traditional Chinese Medicine, Beijing University of Chinese Medicine, Beijing, China; 4 Key Laboratory of Chinese Internal Medicine of Ministry of Education and Beijing Dongzhimen Hospital, Beijing University of Chinese Medicine, Beijing, China

**Keywords:** Jinqiancao granules, liver-gut axis, liver cirrhosis, action mechanism, PI3K-Akt signaling pathway

## Abstract

**Background:**

Current treatments for cirrhosis-a progressive disease marked by fibrosis and inflammation-are limited and non-specific. Jinqiancao granules (JQC), a traditional Chinese medicine used for hepatobiliary diseases, could provide a promising new therapeutic approach.

**Purpose:**

The objective of this study is to explore the therapeutic effects and potential molecular mechanisms of JQC on CCl_4_-induced liver cirrhosis in mice, and to analyze its effects on the liver-gut axis, including liver pathology, the intestinal barrier, and gut microbiota.

**Methods:**

To investigate the therapeutic effects of JQC and its impact on the liver-gut axis, a mouse model of liver cirrhosis was established by intraperitoneal injection of carbon tetrachloride (CCl_4_). The mice were allocated into four groups: the control group (without CCl_4_ induction and receiving normal saline), the model group (CCl_4_-induced and receiving normal saline), and the two treatment groups which received JQC via gavage at either 500 mg/kg (JQC-H) or 250 mg/kg (JQC-L) after CCl_4_ induction. Upon completion of the treatment, all mice were euthanized to collect serum, fecal samples, liver, and intestinal tissues for subsequent analysis. The assessments encompassed histopathology (H&E, Masson, Picro Sirius Red), immunohistochemistry, immunofluorescence, molecular biology (ELISA, Western blot, qRT-PCR), transcriptomics, and 16S rRNA sequencing.

**Results:**

JQC ameliorated CCl_4_-induced liver cirrhosis in mice by improving liver function and suppressing fibrosis, inflammation, and oxidative stress. Mechanistically, it modulated the PI3K-AKT pathway and restored gut-liver axis homeostasis. This was evidenced by intestinal barrier repair (including upregulation of ZO-1 and occludin, and reduction of LPS) and correction of microbial dysbiosis, specifically enriching beneficial bacteria, such as Bacteroidota and Akkermansia, that correlated negatively with liver injury.

**Conclusion:**

This study demonstrates that JQC mitigates liver cirrhosis in mice by modulating the gut-liver axis, enhancing the intestinal barrier, and inhibiting the PI3K-AKT signaling pathway. These results propose JQC as a promising therapeutic candidate, warranting further clinical translation.

## Graphical Abstract

**Figure:**
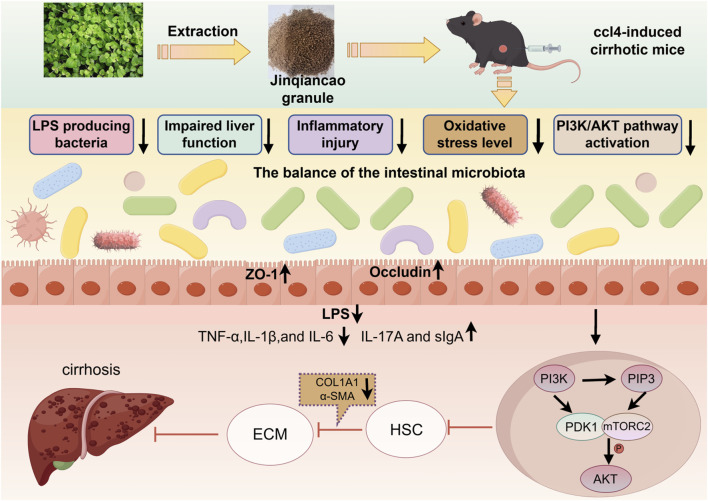

## Introduction

Cirrhosis is a prevalent disease that results in elevated morbidity and mortality rates on a global scale, with its global burden increasing at a steady rate. Epidemiological data indicate that cirrhosis is the 11th leading cause of death globally, resulting in approximately 2 million annual fatalities. This figure constitutes 2.4% of all deaths worldwide, underscoring its serious threat to human health (Juanola et al., 2025). This trend is mirrored in China, where cirrhosis remains highly prevalent. National guidelines report that nearly 300 million people are affected by liver disease, with cirrhosis-related mortality contributing to 11% of the global burden (Chinese Society of Gastroenterology, 2024). Clinically, compensated cirrhosis often presents asymptomatically, leading to frequent underdiagnosis until decompensation occurs. As a result, a considerable number of cases progress to the decompensated stage by the time of diagnosis. Decompensated cirrhosis is frequently accompanied by a variety of serious complications, which not only significantly increase the risk of mortality but also result in a decline in patients’ ability to care for themselves and a substantial reduction in their quality of life (Huang et al., 2023). Due to the complexity of cirrhosis lesions, there is currently a lack of specific and effective treatments apart from partial etiological and symptomatic treatment. Therefore, new treatment strategies for cirrhosis are urgently needed.

To address this urgent need for new therapies, well-established and reliable animal models are indispensable for preclinical research. Among these,the CCl_4_-induced cirrhosis model is a widely utilized experimental paradigm in research. CCl_4_, a hepatotoxic chemical, generates free radicals upon metabolism, which leads to hepatocyte injury and subsequent fibrosis (Fujii et al., 2010). In experimental settings, CCl_4_ is typically administered via subcutaneous or intraperitoneal injection to induce cirrhosis (Wei et al., 2025). This model is characterized by its simplicity and the rapid induction of pathological processes that closely mimic human cirrhosis, including hepatocyte necrosis, inflammatory response, activation of hepatic stellate cells, and collagen deposition (Fujii et al., 2010). In the CCl_4_-induced cirrhosis model, significant elevations in serum alanine aminotransferase (ALT) and aspartate aminotransferase (AST) levels are observed, reflecting the extent of hepatocyte damage (Bin et al., 2012). Histological examination of the liver reveals hepatocyte necrosis, inflammatory cell infiltration, and collagen fiber deposition, which can be visualized through hematoxylin and eosin (H&E) staining and masson staining (Wei et al., 2025). Moreover, Previous studies have reported an increase in the expression of the hepatic fibrosis markers Collagen I and α-SMA, thereby providing further evidence for the occurrence of hepatic fibrosis (Huang et al., 2022). These findings provide essential references for the establishment of the CCl_4_-induced cirrhosis model and the study of associated biomarkers.

Beyond the direct hepatotoxic effects replicated in this model, research has increasingly focused on its broader systemic implications, particularly the role of the liver-gut axis. Cirrhosis pathogenesis is multifactorial, with the gut-liver axis playing a critically implicated role in disease progression. Under physiological conditions, the intestinal mucosal barrier maintains homeostasis by segregating gut microbiota from host immune cells. When this barrier is compromised, the liver becomes directly exposed to bacteria and their endotoxins, potentially resulting in intestinal dysfunction (Pabst et al., 2023; Ichikawa et al., 2024). In addition, gut dysbiosis can further impair barrier integrity and promote bacterial translocation, leading to infections, systemic inflammation, and vasodilation, ultimately precipitating acute decompensation and organ failure (Trebicka et al., 2021). Consequently, targeting the gut-liver axis represents a potential therapeutic avenue for cirrhosis.

Moreover, traditional Chinese medicine (TCM) has exhibited notable benefits in the treatment of cirrhosis, and its efficacy is garnering mounting recognition. This phenomenon can be attributed to the drug’s multi-component, multi-target, and multi-pathway mechanisms of action and characteristics (Zheng et al., 2025). Research has demonstrated that TCM can inhibit cirrhosis progression by concurrently ameliorating inflammatory responses, oxidative stress, and fibrosis, thereby underscoring its multi-targeted therapeutic potential (Wei et al., 2022).

Jinqiancao granules (JQC) are a single-ingredient preparation derived from the dried whole plant of Lysimachia christinae Hance, a species of the genus Lysimachia in the family Primulaceae. The product has been approved by the China National Medical Products Administration (approval number: Z20027654). The preparation process involves the dispersion of JQC extract, the incorporation of suitable pharmaceutical excipients, and the addition of a sufficient amount of flavor enhancer. Subsequently, a vacuum low-temperature continuous drying machine is employed to produce a mixed dry extract powder, which is then mixed with ethanol and stirred to form granules (patent number: CN201710157190.1). JQC is a widely used herbal medicine for treating liver and gallbladder diseases (Liu et al., 2021; Deng et al., 2015), with effects including clearing heat, promoting diuresis, reducing swelling, and alleviating inflammation. However, the potential mechanism of JQC in treating liver cirrhosis remains unclear. Therefore, this study aims to explore the mechanism of action of JQC against CCl_4_-induced liver cirrhosis based on the liver-gut axis.

## Materials and methods

### Main materials and reagents

JQC (Dikang Pharmaceutical Co. Ltd., Chengdu, China); Olive oil (MACKLIN Company, Shanghai, China); 10% carbon tetrachloride (10% CCl_4_) (MACKLIN Company, Shanghai, China); anti-GAPDH (1:50, AF7021, Rabbit, Affinity, Jiangsu, China); anti-alpha-SMA (1:50, 55135-1-AP, Rabbit, Proteintech, Wuhan, China); anti-CollagenI (1:50,86093-1-RR, Rabbit, Proteintech, Wuhan, China); anti-occludin (1:50, DF7504, Rabbit, Affinity, Jiangsu, China); anti-ZO-1 (1:50, AF5145, Rabbit, Affinity, Jiangsu, China); anti-AKT (1:50, 10176-2-AP, Proteintech, Rabbit, Wuhan, China); anti-p-AKT (1:50, 66444-1-Ig, Mouse, Proteintech, Wuhan, China); anti-PI3K、anti-p-PI3K (1:50, #4249、#4228, Rabbit, Cell Signaling, United States); ProteinSimple Jess Western Kit (PS-ST01EZ, BioTechne, Shanghai, China); RIPA Lysis Buffer (PC101, Epizyme Biomedical Technology Co., Ltd., Shanghai, China); BCA Protein Concentration Assay Kit (PC0020, Solarbio, Beijing, China); All-in-One First-Strand SuperMix、RaPure Total RNA Kit (MD80101、R4011-01, Magen, Guangzhou, China); Hematoxylin-Eosin (H&E) Stain Kit (G1004, Servicebio, Wuhan, China); Masson Stain Kit (G1006, Servicebio, Wuhan, China); Immunohistochemistry (IHC) Kit (G1211, Servicebio, Wuhan, China); EDTA Antigen Retrieval Solution (B2001, Edison Biotechnology Co., Ltd., Jiangsu, China); Citric Acid Antigen Retrieval Solution (B2010, Edison Biotechnology, Jiangsu, China); Autofluorescence Quencher (B0008, Edison Biotechnology, Jiangsu, China); Picro Sirius Red Stain (PSR) Kit (B1068, Edison Biotechnology Co., Ltd., Jiangsu, China).

### Laboratory animals

SPF grade C57BL/6J mice (n = 24) with a weight range of (20 ± 2) grams were procured from Beijing Vital River Laboratory Animal Technology Co., Ltd. The laboratory animal production license number is SCXK (Beijing) 2021-0006. All mice were housed at the Beijing University of Chinese Medicine Laboratory Animal Center. The mice were maintained under a 24-h light/dark cycle, with controlled temperature maintained at (22 ± 3)°C and relative humidity at (50 ± 10)%. All mice had unrestricted access to food and water, and experiments were conducted after a 1-week acclimatization period. This experimental protocol was reviewed and endorsed by the Beijing University of Chinese Medicine Experimental Animal Welfare and Ethics Review Committee (No. BUCM-2024091003-3277).

### Construction and administration of CCl_4_-induced liver cirrhosis mouse models

Mice were randomly assigned to four groups (n = 6): the control group, the model group, the JQC high-dose (JQC-H,human equivalent dose, 500 mg/kg/day) group (Reagan-Shaw et al., 2008), and the JQC low-dose (JQC-L, half of the high dose, 250 mg/kg/day) group. A liver cirrhosis model was induced in all but the control group via twice-weekly intraperitoneal injections of CCl_4_-olive oil mixture (1:9) for 14 weeks. From week 9, the following 6-week gavage treatments were administered: the control group (non-induced) and the model group (CCl_4_-induced) received a corresponding volume of normal saline, the JQC-H and JQC-L groups (CCl_4_-induced) received Jinqiancao granules at 500 mg/kg/d and 250 mg/kg/d, respectively. Upon study completion, samples (serum, feces, liver, and intestine) were collected after euthanasia.

### Serum biochemical indicators

Serum biochemical indicators, including alanine transaminase (ALT), aspartate aminotransferase (AST), total bilirubin (TBIL), albumin (ALB), and total bilirubin (TBIL), were measured using the Beckman Coulter AU biochemical analyzer (Beckman Coulter, California, United States).

### The enzyme-linked immunosorbent assay (ELISA)

We examined hyaluronic acid (HA, MB3250414), interleukin-6 (IL-6, MB28990414), interleukin-1β (IL-1β, MB27760122), interleukin-17A (IL-17A, MB58180122), tumor necrosis factor-α (TNF-α, MB28680122), superoxide dismutase (SOD, MB31250122), reduced glutathione (GSH, MB56130122), and secretory immunoglobulin A (sIgA, MB31660122). Lipopolysaccharide/endotoxin (LPS, MB34180122) levels were detected using specific ELISA kits from Meibiao Biotechnology in Jiangsu, China. The kits were used according to the instructions in the manual.

### Histopathological staining and observation

Liver or intestinal tissue samples from mice were fixed in 4% paraformaldehyde, embedded in paraffin, and sectioned. The sections were then subjected to H&E, Masson, and PSR staining to observe morphological changes and the extent of fibrosis, as described in previous studies (Wang et al., 2024; Fang et al., 2022).

### Immunohistochemistry (IHC)

After routine deparaffinization and rehydration, liver or intestinal sections were subjected to immunohistochemical staining for Col1a1, α-SMA, ZO-1, and occludin, following the manufacturer’s protocols, as previously described (Qi et al., 2025). Ultimately, the positively stained areas were quantified and evaluated using ImageJ software.

### Immunofluorescence (IF)

After deparaffinization and antigen retrieval, we quenched autofluorescence for 5 min, blocked with BSA, and incubated sections with primary antibodies at 4 °C overnight. We then applied species-matched secondary antibodies in the dark at room temperature, counterstained nuclei with DAPI, and acquired images for microscopic analysis.

### Liver transcriptomics sequencing

Total RNA was extracted from liver tissues of control, model, and JQC-treated mice (n = 3 per group) using TRIzol® reagent (Thermo Fisher Scientific, United States), following the manufacturer’s protocol. RNA quality was assessed using the Agilent 5300 Bioanalyzer, and concentration was measured with the ND-2000 system (NanoDrop Technologies). Subsequent steps including RNA purification, reverse transcription, and transcriptome library construction were conducted by Shanghai Major Bio-Biopharm Biotechnology Co., Ltd. (Shanghai, China). Libraries were prepared using the Illumina® Stranded mRNA Prep, Ligation Kit (San Diego, CA) with 1 μg of total RNA as input. For transcriptome analysis, gene abundance was quantified using RSEM, and differential expression analysis was performed with DESeq2 or DEGseq. Differentially expressed genes (DEGs) were defined as those with |log_2_FC| ≥ 1 and FDR <0.05 (DESeq2) or FDR <0.001 (DEGseq). Functional annotation of therapeutic targets was conducted using the DAVID database for GO and KEGG enrichment analyses. Gene set enrichment analysis (GSEA) was further applied to evaluate expression disparities in key signaling pathways across the control, model, and JQC groups.

### JQC’s network pharmacology analysis

The chemical composition of Jinqiancao granules (JQC) was identified based on literature review and HPLC-ESI-Q-TOF-MS analysis (Wang et al., 2016). Potential active components and their corresponding targets were retrieved from the PubChem and SwissTarget Prediction databases. Cirrhosis-related targets were collected from the GeneCards, OMIM, and TTD databases. The overlapping targets between JQC and cirrhosis were subsequently identified. A protein-protein interaction (PPI) network was constructed using the STRING database and visualized in Cytoscape 3.10.3. Core targets were screened with the cytoNCA plugin. Finally, Gene Ontology (GO) and Kyoto Encyclopedia of Genes and Genomes (KEGG) pathway enrichment analyses were performed on the potential targets using the DAVID database.

### Quantitative real-time PCR (qRT-PCR)

Total RNA was extracted from liver tissues from mice in each group using a RaPure Total RNA kit. The extracted RNA was reverse transcribed into cDNA using All-in-One First-Strand SuperMix. Next, qRT-PCR was performed using the Agilent Technologies Real-Time PCR Detection System and Magen Universal SYBR qPCR Mix. The primers and their detailed information, including sequences and product sizes, are provided in Supplementary Table S1.

### Protein expression detection

Liver tissues from each group were homogenized and lysed on ice using RIPA buffer supplemented with protease and phosphatase inhibitors. The homogenates were ground using a low-temperature grinder for 5 min, followed by centrifugation at 12,000 × g for 10 min at 4 °C. The resulting supernatant was collected, and protein concentration was determined using a BCA assay kit. Protein expression levels were analyzed on an automated capillary Western blot system (ProteinSimple, Bio-Techne). Primary antibodies against PI3K, p-PI3K, AKT, p-AKT, and GAPDH were used at a 1:50 dilution. All other reagents were included in the ProteinSimple kit, and the procedure was conducted in accordance with the manufacturer’s instructions. Data were analyzed with Compass for SW software.

### High-throughput 16S rRNA sequencing

Total microbial genomic DNA was extracted from fecal samples of C57BL/6J mice using the FastPure Stool DNA Isolation Kit (MJYH, Shanghai, China). DNA quality and concentration were assessed by 1.0% agarose gel electrophoresis and a NanoDrop 2000 spectrophotometer (Thermo Scientific, United States), and samples were stored at −80 °C until use. The V3-V4 hypervariable region of the bacterial 16S rRNA gene was amplified with the primers 338F (5′-ACT​CCT​ACG​GGA​GGC​AGC​AG-3′) and 806R (5′-GGACTACHVGGGTWTCTAAT-3′) on a T100 Thermal Cycler (Bio-Rad, United States). The resulting PCR amplicons were purified using a DNA gel recovery kit (YuHua, Shanghai) and quantified with a Qubit 4.0 fluorometer (Thermo Fisher Scientific, United States). After quantification, libraries were constructed using the NEXTFLEX Rapid DNA-Seq Kit and sequenced on the Illumina NextSeq 2000 platform at Majorbio Bio-Pharm Technology Co., Ltd. (Shanghai, China).

### Statistical analysis

All data are presented as mean ± standard deviation (SD). GraphPad Prism 10.0 was used for all statistical analyses and graphing. Data were first assessed for normality using the Shapiro-Wilk test and for homogeneity of variances using the Brown-Forsythe test. For data meeting these parametric assumptions, one-way ANOVA was performed, followed by Fisher’s LSD *post hoc* test for inter-group comparisons. For data violating these assumptions, the non-parametric Kruskal–Wallis test was applied, followed by Dunn’s *post hoc* test. P values <0.05 were considered statistically significant.

## Results

### The effect of JQC on liver damage in CCl_4_-induced cirrhotic mice

**FIGURE 1:**
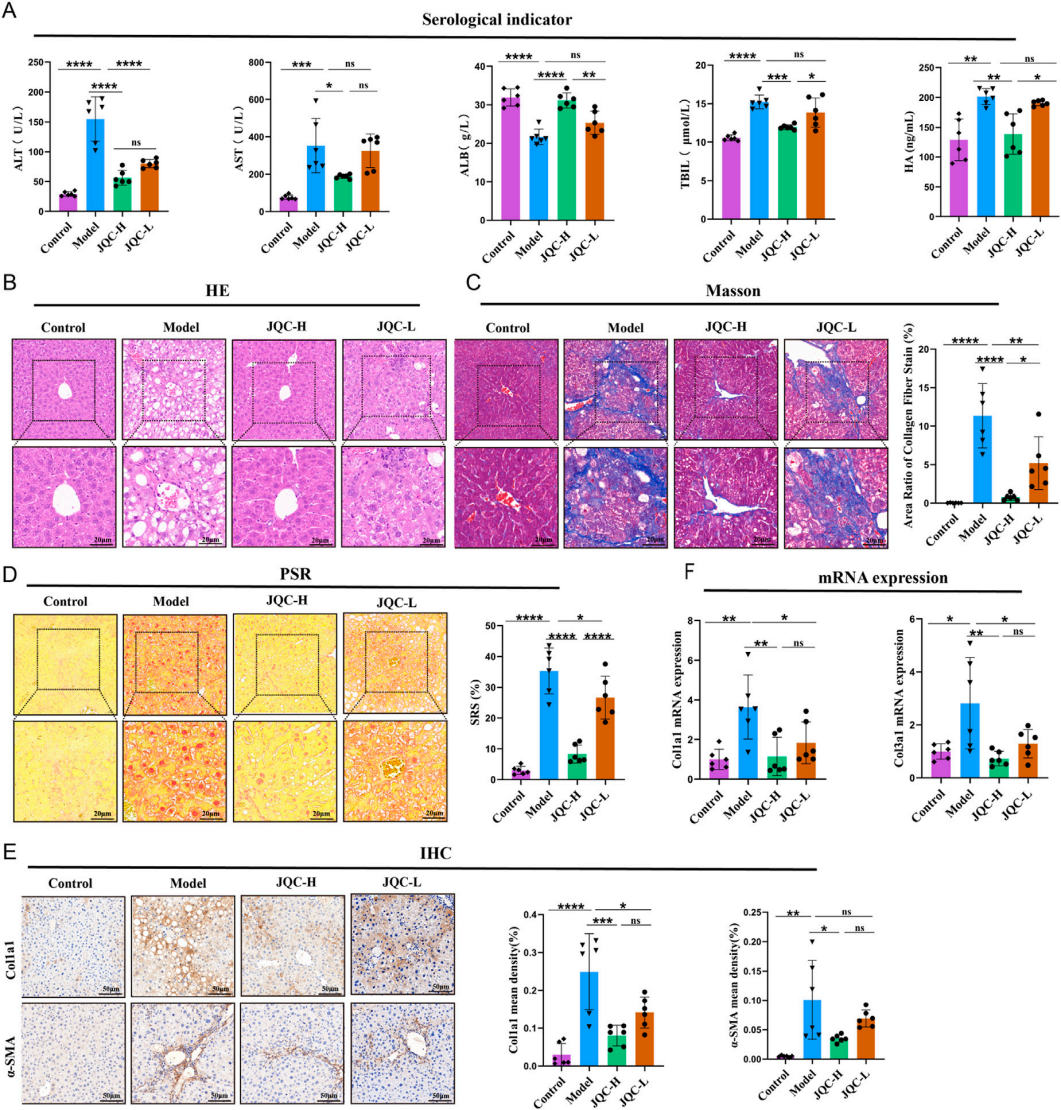
Effect of JQC on liver injury in mice with CCl4-induced cirrhosis. **(A)** Detection of serological indicators in mice. **(B)** HE staining of mouse liver (20 × & × 40 magnification). **(C)** Masson staining of mouse liver (20 × & × 40 magnification) and the area of collagen fibre staining. **(D)** PSR staining of mouse liver (20 × & × 40 magnification). **(E)** Mouse IHC staining of Col1a1 and a-SMA proteins in liver (×20 magnification). **(F)** mRNA expression level of Col3a1. Data are expressed as mean ± SD. *p < 0.05, **p < 0.01, ***p < 0.001, ****p < 0.0001, ns, not significant.

Serum results (Figure 1A) showed that compared to the control group, the model group had significantly elevated levels of ALT, AST, TBIL, and HA, but a significantly reduced level of ALB. JQC-H intervention improved these changes.

To further investigate the recovery of hepatic pathological tissue damage, H&E staining, masson staining, and PSR staining were employed. H&E staining revealed that the liver lobule structure in the control group mice remained normal, with hepatocytes exhibiting regular radial arrangement and no evidence of inflammatory cell infiltration or necrosis. In contrast, the liver tissue structure in the model group mice showed significant abnormalities, including the partial formation of pseudo-lobules, connective tissue proliferation, and inflammatory cell infiltration, findings that are consistent with those reported in previous studies (Wang et al., 2024; Fang et al., 2022). Following JQC intervention, pathological changes in the liver were alleviated to varying degrees across all groups (Figure 1B). Masson and PSR staining (Figures 1C,D) revealed marked collagen fiber deposition in the model group. JQC intervention resulted in a dose-dependent reduction of this fibrosis, with the JQC-H group showing superior efficacy, consistent with the trend observed in HE staining.

To further assess liver fibrosis, we performed IHC. The results revealed a significant increase in the protein expression of COL1A1 and α-SMA in the model group compared to the control group (Figure 1E). This finding is consistent with prior report (Shen et al., 2022). JQC treatment reduced their expression in a dose-dependent manner.

This anti-fibrotic effect was corroborated at the mRNA level, where JQC likewise dose-dependently downregulated the model-induced overexpression of Col1a1 and Col3a1 (Figure 1F). Consequently, based on the comprehensive results, the high dose of JQC was selected for the subsequent experiments.

### The effect of JQC on the transcriptomic profile of CCl_4_-induced cirrhotic mice

**FIGURE 2:**
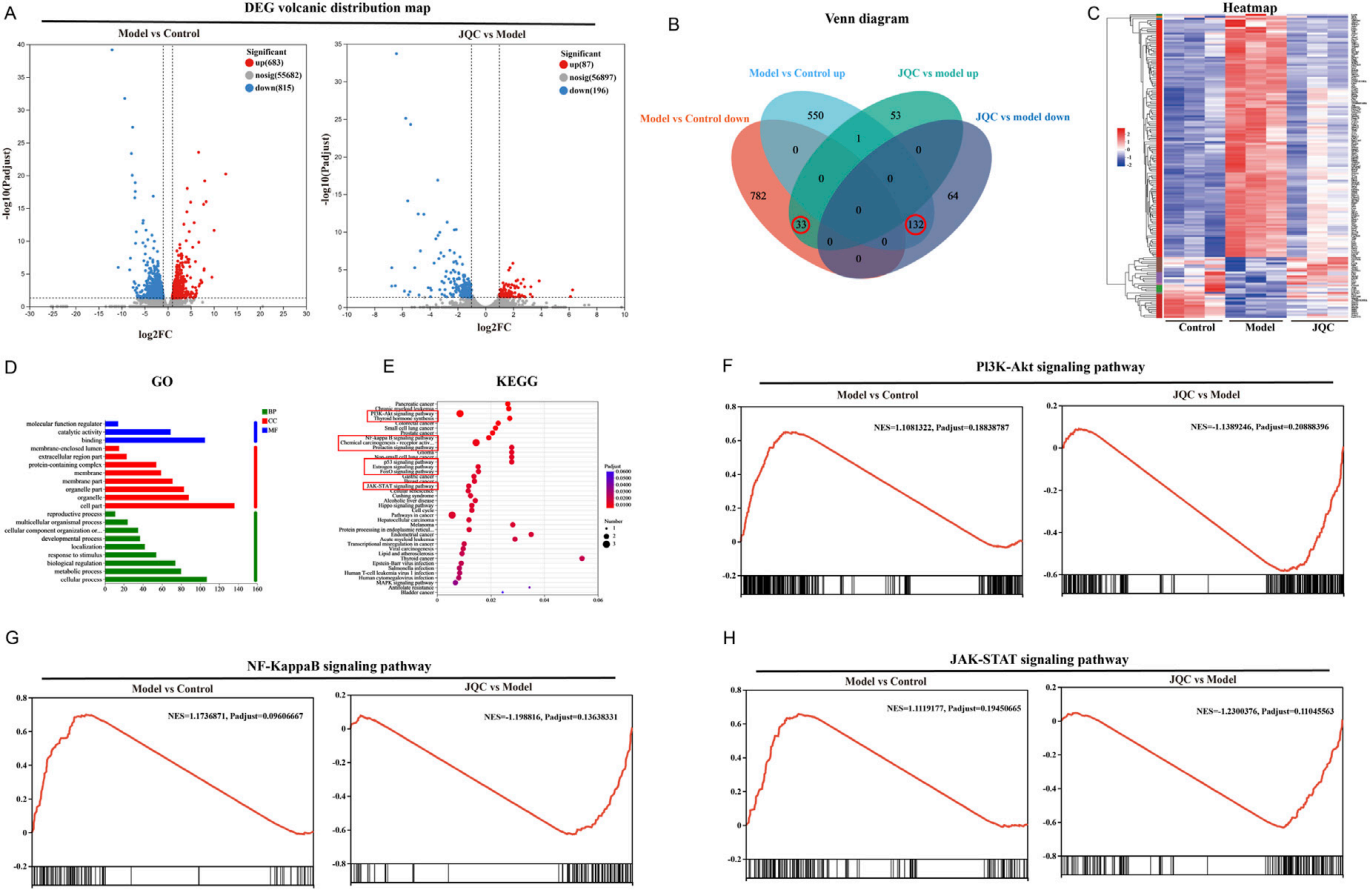
Screen the key genes related to the treatment of JQC against liver cirrhosis. **(A)** Distribution map of DEG volcanoes. Blue dots show downregulation of genes, while red dots show upregulation. **(B)** Intersection results of DEGs for the control, model and JQC groups. **(C)** Heatmap of expression of therapeutic genes. **(D)** GO annotation. **(E)** KEGG pathway enrichment analysis. **(F)** GSEA results for the PI3K-AKT signalling pathway. **(G)** GSEA results for the NF-kappa B signalling pathway. **(H)** GSEA results for the JAK-STAT signalling pathway.

Transcriptomic analysis of liver tissues from control, model, and JQC-treated mice identified differentially expressed genes (DEGs) under the threshold of |fold change| ≥2 and adjusted p-value <0.05. A total of 1,498 DEGs were detected between the control and model groups, including 683 upregulated and 815 downregulated genes. Between the model and JQC groups, 283 DEGs were identified, of which 87 were upregulated and 196 downregulated (Figures 2A,B). By comparing these DEGs, 165 genes potentially associated with the therapeutic effect of JQC on cirrhosis were obtained (Figure 2B). The expression patterns of these treatment-related genes across samples are visualized in the clustering heatmap shown in Figure 2C.

To elucidate the potential biological mechanisms of JQC against cirrhosis, the therapeutic target genes were subjected to GO annotation and KEGG pathway enrichment analysis using the DAVID database. This approach was undertaken to elucidate the potential biological pathways underlying the efficacy of JQC treatment in addressing cirrhosis. GO results demonstrated that these therapeutic genes were significantly enriched in 18 biological processes (BP), 14 cellular components (CC), and 11 molecular functions (MF) (*p* < 0.05). BP encompassed three distinct categories: molecular function regulator, catalytic activity, and binding. Concerning the CC category, the therapeutic genes were predominantly enriched in the following categories: cell part, organelle, organelle part, membrane part, membrane, protein-containing complex, extracellular region part, and membrane-enclosed lumen. MF primarily included cellular processes, metabolic processes, biological regulation, response to stimulus, localization, developmental processes, cellular component organization or biogenesis, multicellular organismal processes, and reproductive processes. The figure illustrates the distribution of highly enriched GO terms in BP, CC, and MF, as determined by the ranking.

KEGG enrichment analysis identified 165 signaling pathways in total, with the top 40 pathways displayed in Figure 2E. After excluding those related to disease conditions, nine key pathways were selected as potentially central to JQC’s therapeutic effect. These include the PI3K-Akt, NF-κB, FoxO, JAK-STAT, p53, prolactin, estrogen, and thyroid hormone signaling pathways, as well as chemical carcinogenesis–receptor activation (Figure 2E).

Gene set enrichment analysis (GSEA) was further performed to evaluate the expression patterns of the nine candidate KEGG pathways across the control, model, and JQC groups. Results identified three pathways as being significantly modulated by JQC treatment: PI3K-AKT, NF-κB, and JAK-STAT (|NES| > 1, FDR < 0.25). As summarized in Table 1, these pathways were significantly upregulated in the model group compared to the controls (NES > 0, FDR < 0.25), whereas their activity was markedly suppressed in the JQC-treated group (NES < 0, FDR < 0.25). The corresponding GSEA plots are presented in Figures 2F–H, collectively supporting the role of these three signaling pathways as central mechanisms underlying the therapeutic effect of JQC on cirrhosis.

**TABLE 1:** Three KEGG signaling pathways identified by GSEA.

ID	Description	Model group vs. Control group		Model group vs. JQC group	
		NES	Padjust	NES	Padjust
MMU04151	PI3K-AKT	1.1081322	0.18838787	−1.1389246	0.20888396
MMU04064	NF-kappa B	1.1736871	0.09606667	−1.198816	0.13638331
MMU04630	JAK-STAT	1.1119177	0.19450665	−1.2300376	0.11045563

### Network pharmacology analysis of JQC for the treatment of liver cirrhosis

**FIGURE 3:**
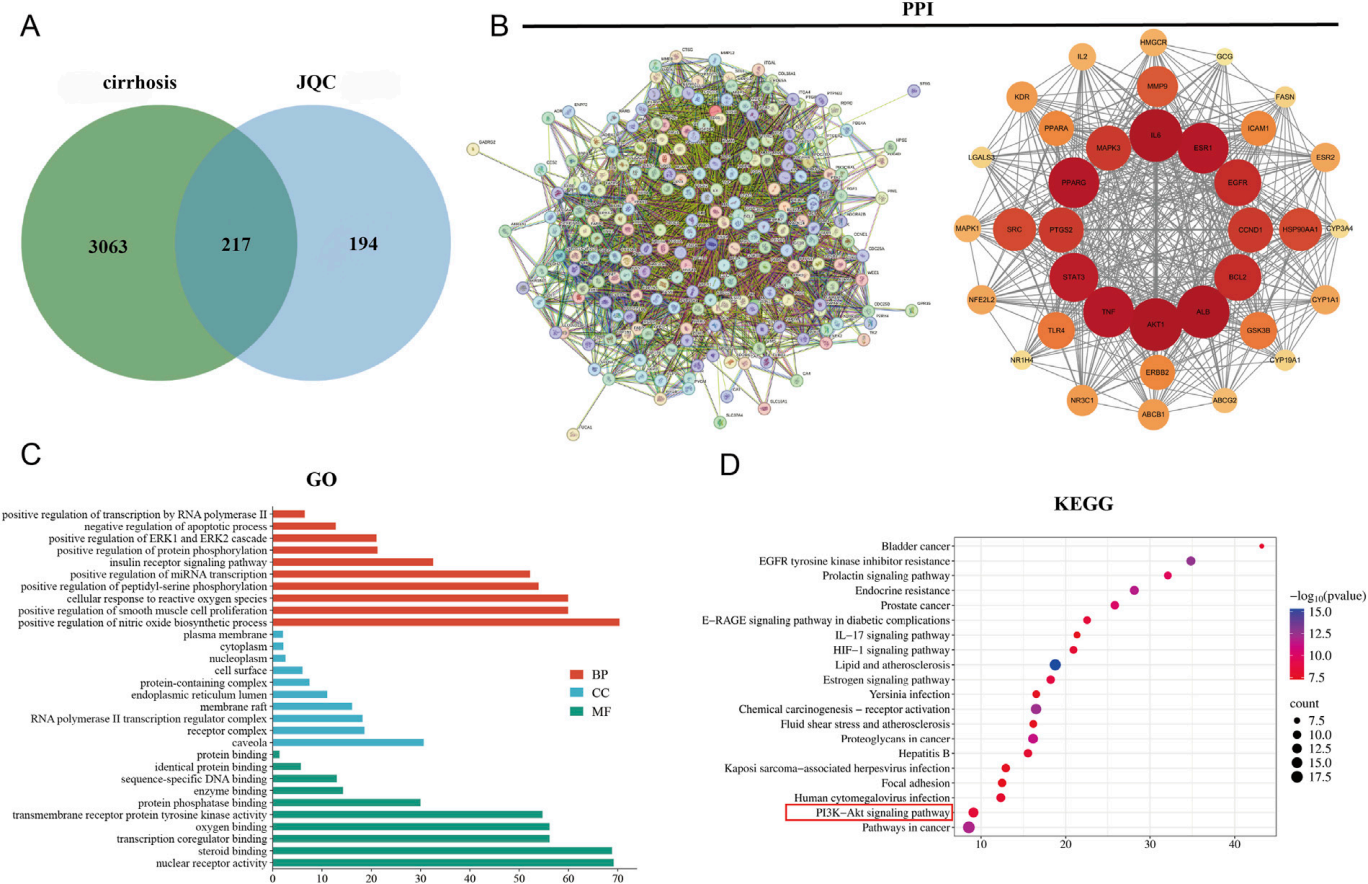
Potential targets and enrichment analysis of JQC against cirrhosis. **(A)** Cirrhosis and JQC target Venn plots. **(B)** Construction of the PPI network map for intersecting targets. **(C)** GO enrichment analysis. **(D)** KEGG enrichment analysis.

Based on literature-derived fingerprint data, 31 chemical components of JQC were identified, including sucrose, uridine, gallic acid, and other phenolic acids and flavonoids (Wang et al., 2016). A total of 415 potential protein targets of these components were retrieved from PubChem and the SwissTarget Prediction database. Meanwhile, 3,280 cirrhosis-related targets were collected from the GeneCards, OMIM, and TTD databases. Intersection of the drug and disease targets yielded 217 shared targets, as depicted in the Venn diagram (Figure 3A).

The 217 overlapping target genes were imported into the STRING database to construct a PPI network. After removing unconnected nodes, the network was analyzed in Cytoscape. Topological features including degree centrality (DC), betweenness centrality (BC), and closeness centrality (CSC) were calculated using the CytoNCA plugin. Targets with BC, CSC, and DC values above or equal to the median were retained, leading to the identification of 36 core target genes (Figure 3B). The top 10 hub genes, ranked by degree value, were AKT1 (degree = 141), IL6 (degree = 139), ALB (degree = 135), TNF (degree = 133), STAT3 (degree = 112), EGFR (degree = 112), ESR1 (degree = 110), SRC (degree = 108), BCL2 (degree = 105), and PPARG (degree = 97).

The core target genes were subjected to further GO and KEGG enrichment analyses. In the GO analysis, a total of 220 functional terms were significantly enriched, including 139 biological process (BP) terms—such as positive regulation of transcription by RNA polymerase II, negative regulation of apoptotic process, and positive regulation of the ERK1 and ERK2 cascade—20 CC terms, primarily encompassing caveola, receptor complex, and RNA polymerase II transcription regulator complex, and 61 MF terms, mainly involving nuclear receptor activity, steroid binding, and transcription coregulator binding. The top 10 most significantly enriched GO terms in each category are displayed in Figure 3C.KEGG pathway analysis identified the top 20 pathways associated with the therapeutic effects of JQC on cirrhosis (Figure 3D). Among these, the PI3K–AKT signaling pathway was recognized as a key pathway. Notably, this pathway aligns closely with the core signaling mechanisms implicated in JQC’s anti-cirrhotic action, as previously revealed by transcriptomic data.

### The effect of JQC on the PI3K-AKT signaling pathway in CCl4-induced cirrhotic mice

**FIGURE 4:**
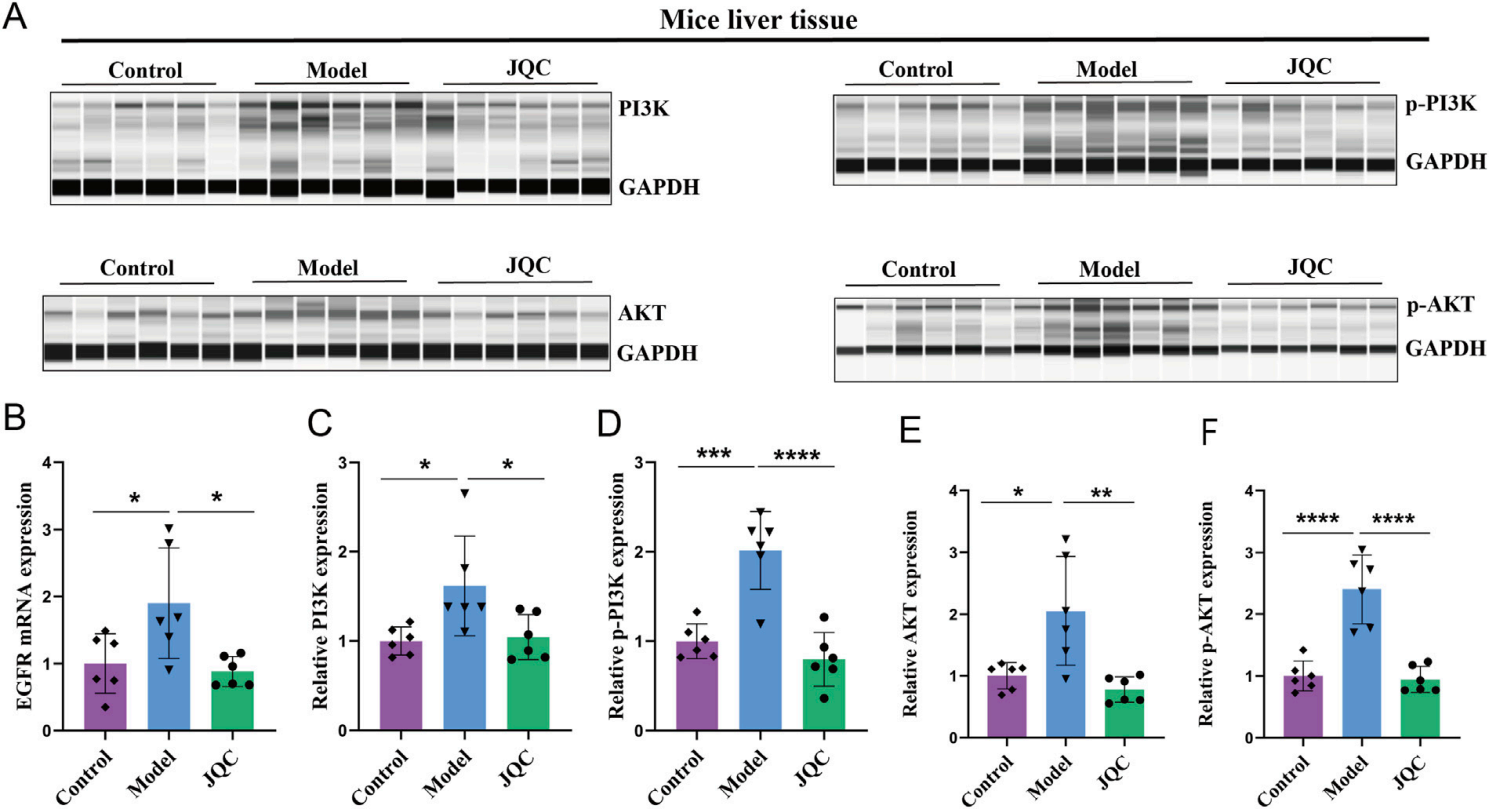
The Effect of JQC on the PI3K-AKT Signaling Pathway in CCl_4_-Induced Cirrhotic Mice. **(A)** Bar graphs of total PI3K and AKT proteins and phosphorylated proteins. **(B)** The mRNA expression of EGFR. **(C)** PI3K protein expression in mice liver tissue. **(D)** p-PI3K protein expression in mice liver tissue. **(E)** AKT protein expression in mice liver tissue. **(F)** p-AKT protein expression in mice liver tissue. Data are expressed as mean ± SD. *p < 0.05, **p < 0.01, ***p < 0.001, ****p < 0.0001.

Using transcriptomics data and network pharmacology to screen environmental information, we identified the PI3K-AKT signaling pathway, containing the core protein AKT1, as the top-ranked pathway. Therefore, we conducted follow-up experiments to verify JQC’s effect on the PI3K-AKT signaling pathway. The results demonstrated in Figure 4 revealed that within the model group, the mRNA expression levels of EGFR, a pivotal upstream signaling molecule in this pathway, along with the pathway-related proteins PI3K, p-PI3K, AKT, and p-AKT, exhibited an upward trend. Concurrently, JQC significantly curtailed the expression levels of these proteins and the EGFR gene. In sum, these findings offer significant insights into the mechanism of action of JQC in the treatment of cirrhosis. It has been demonstrated that the modulation of the PI3K-AKT signaling pathway can improve the progression of cirrhosis.

### The effect of JQC on intestinal barrier damage in CCl_4_-induced cirrhotic mice

**FIGURE 5:**
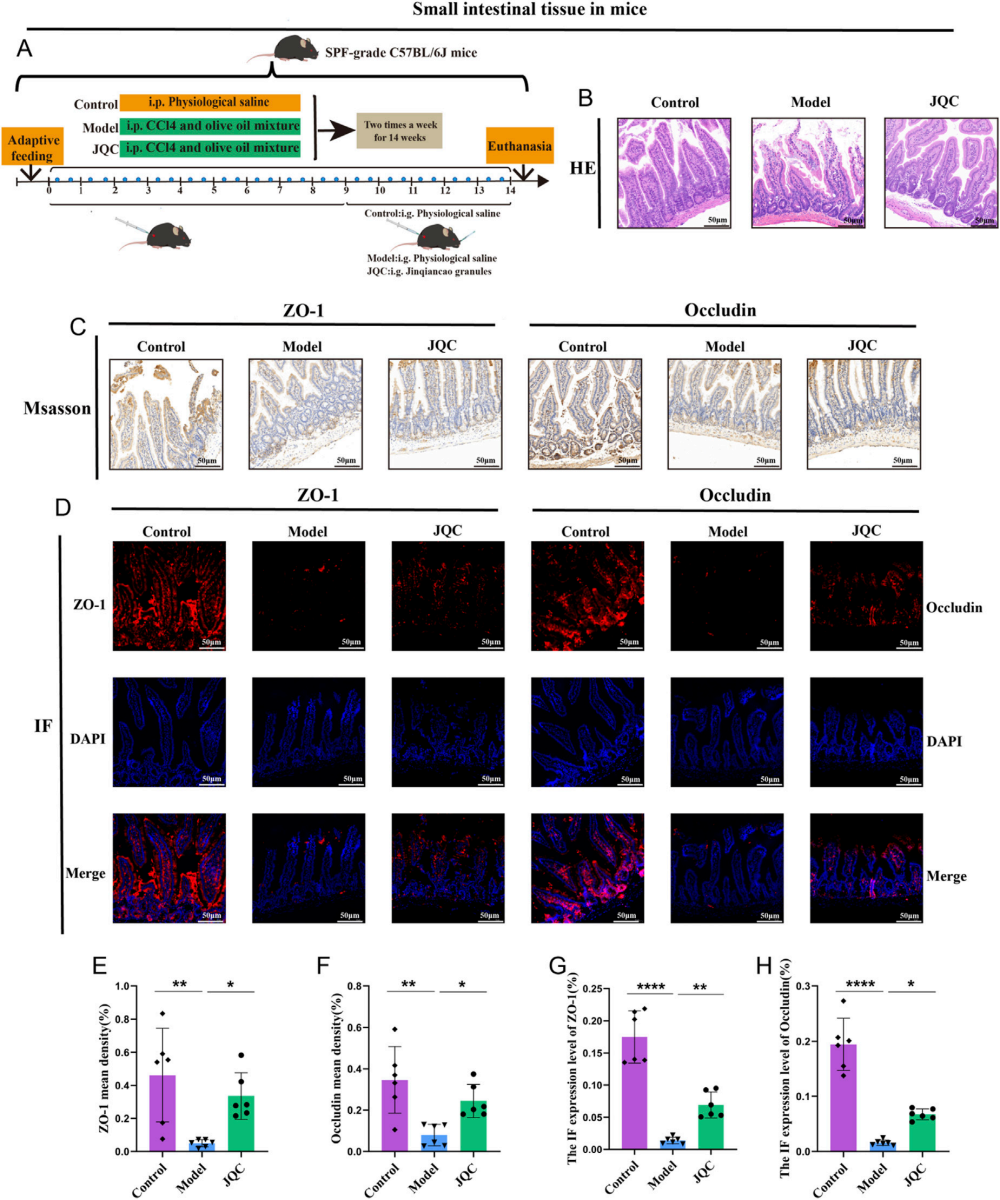
The Effect of JQC on CCl4-Induced Intestinal Barrier Damage in Cirrhotic Mice. **(A)** Scheme and design. **(B)** HE staining of small intestinal tissue. **(C)** ZO-1 and Occludin protein expression in small intestinal tissues. **(D)** Fluorescent expression of ZO-1 and Occludin proteins in small intestinal tissues. **(E)** ZO-1 mean density. **(F)** Occludin mean density. **(G)** Statistical graph of protein fluorescence expression of ZO-1. **(H)** Statistical graph of protein fluorescence expression of Occludin. Data are expressed as mean ± SD. *p < 0.05, **p < 0.01, ****p < 0.0001.

Histological examination of the small intestine revealed an intact tissue architecture with neatly arranged epithelial cells and no signs of detachment, crypt dilation, or inflammatory infiltration in control mice. In contrast, the model group displayed severe mucosal damage, including villous epithelial detachment, lamina propria exposure, and inflammatory cell infiltration. JQC intervention effectively ameliorated these pathological alterations (Figure 5B). Moreover, the expression levels of intestinal tight junction proteins ZO-1 and occludin, as assessed by both Western blot and immunofluorescence, were markedly downregulated in CCl_4_-induced cirrhotic mice compared with controls, whereas JQC treatment restored their expression (Figures 5C–H). These results indicate that JQC contributes to the repair and protection of the intestinal mucosal barrier in experimental cirrhosis.

### The effect of JQC on the inflammatory response and oxidative stress levels in CCl_4_-induced cirrhotic mice

**FIGURE 6:**
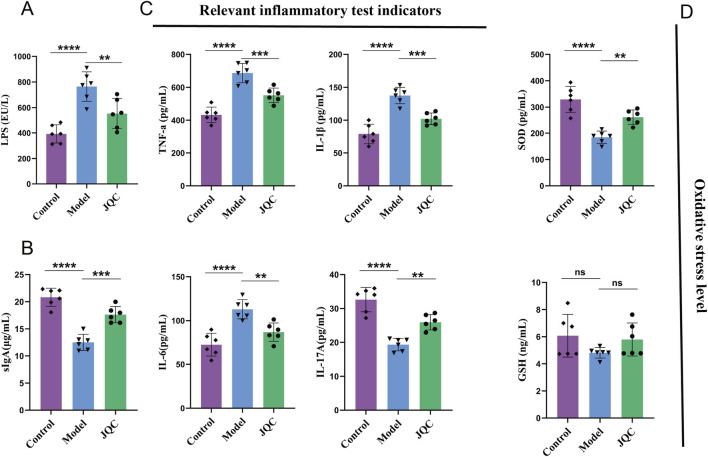
The effect of JQC on the inflammatory response and oxidative stress level in CCl4-induced cirrhotic mice. **(A)** LPS levels in mouse serum. **(B)** sIgA levels in mouse intestinal tissues. **(C)** Inflammation-related indices in mouse liver tissues. **(D)** Oxidative stress-related indices in mouse liver tissues. Data are expressed as mean ± SD. **p < 0.01, ***p < 0.001, ****p < 0.0001, ns: not significant.

LPS plays a critical role in triggering hepatic inflammatory responses (An et al., 2022). When the intestinal barrier is impaired, LPS translocates into the lamina propria, activating the immune system and promoting the release of pro-inflammatory cytokines such as TNF-α and IL-6, which in turn exacerbate intestinal barrier damage. In the present study, serum LPS levels were significantly elevated in the model group compared with the control group (Figure 6A), indicating severe intestinal barrier disruption in CCl_4_-induced cirrhotic mice. Notably, JQC treatment effectively attenuated this pathological progression.

Secretory immunoglobulin A (sIgA) serves as a critical component of intestinal mucosal immunity and gut-liver axis communication. Impairment of the intestinal barrier reduces sIgA secretion, facilitating the translocation of gut microbiota and metabolites into systemic circulation, thereby exacerbating liver injury (Fukui, 2021). Consistent with this mechanism, sIgA levels were markedly decreased in the intestinal tissue of cirrhotic model mice (Figure 6B), indicating compromised mucosal immune defense. Treatment with JQC significantly restored sIgA expression, indicating its ability to reinforce intestinal immunity and maintain barrier integrity.

Inflammatory responses are key drivers in the progression of chronic liver disease. (Qi et al.,2024). To evaluate the impact of JQC on immune damage induced by liver fibrosis, we analyzed the expression levels of various inflammatory factors and inflammation-related substances. The results showed that mice in the CCl_4_-treated model group exhibited significant accumulation of TNF-α, IL-1β, and IL-6, as well as a significant reduction in IL-17A (Figure 6C). However, JQC treatment led to a significant reduction in the expression of TNF-α, IL-1β, and IL-6, accompanied by an increase in IL-17A levels.

Analysis of oxidative stress markers revealed significantly decreased SOD levels in CCl_4_-induced cirrhotic mice compared to controls (Figure 6D). While GSH levels also showed a decreasing trend, the change was not statistically significant. Treatment with JQC notably upregulated SOD expression and slightly increased GSH, though the latter change remained non-significant. These results suggest that JQC alleviates oxidative stress in cirrhotic mice, primarily through the restoration of SOD activity.

### The effect of JQC on CCl_4_-induced intestinal microbiota imbalance in mice with cirrhosis

**FIGURE 7:**
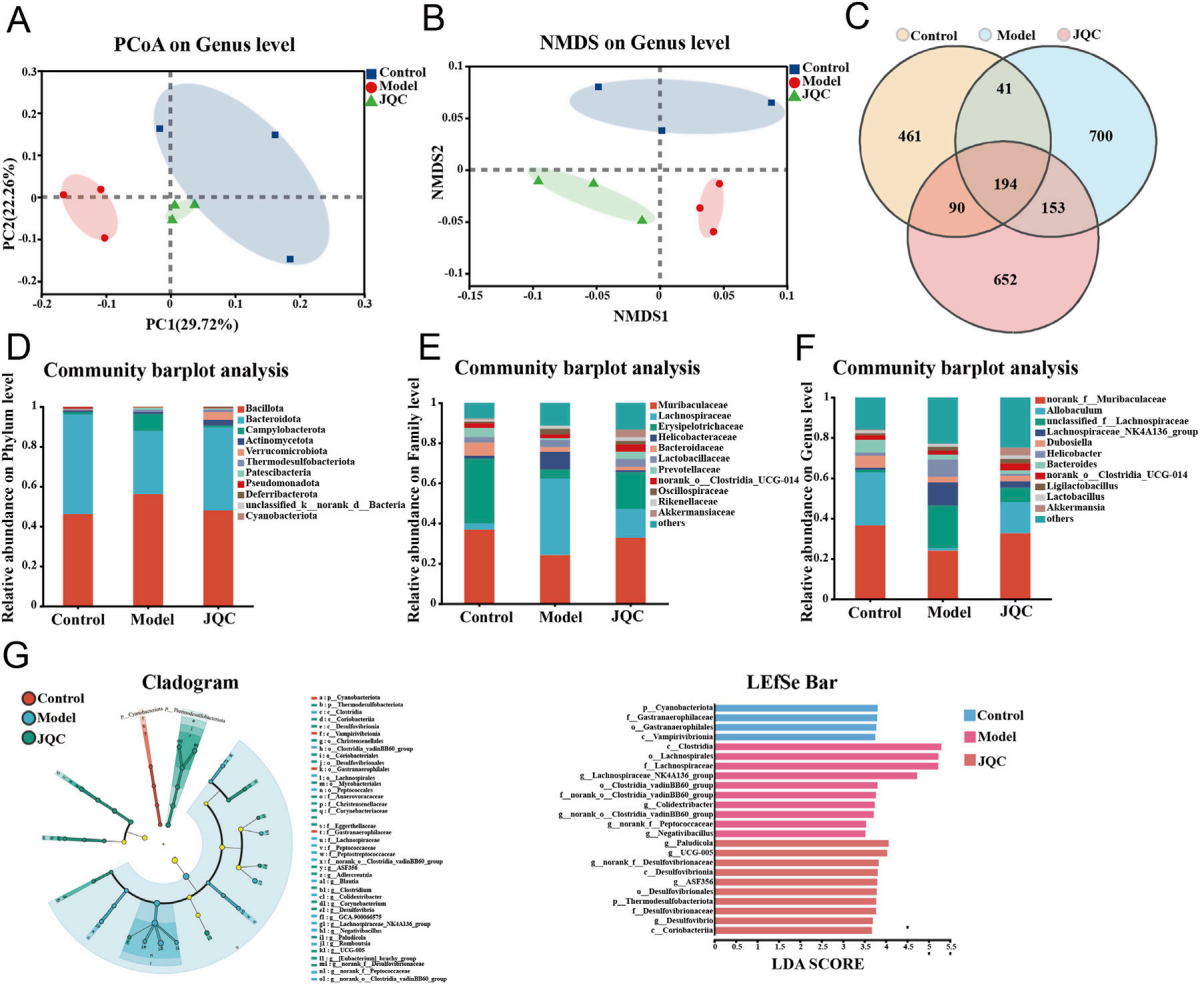
The effect of JQC on intestinal flora dysbiosis in mice with CCl_4_-Induced liver cirrhosis. **(A)** PCoA for each group. **(B)** NMDS for each group. **(C)** OTU Venn diagram. **(D)** Alterations of the intestinal flora comparison at the phylum level. **(E)** Alterations of the intestinal flora comparison at the family level. **(F)** Alterations of the intestinal flora comparison at the genus level. **(G)** The major species in the intestinal flora of the three groups of mice were identified using LEfSe analysis.

Gut microbiota dysbiosis has been reported to compromise intestinal mucosal barrier integrity (An et al., 2022). In this study, we employed 16S rRNA sequencing to profile the gut microbiota composition across the three mouse groups. Beta diversity, assessed by both principal coordinate analysis (PCoA) and non-metric multidimensional scaling (NMDS), revealed clear structural separation among the groups. Specifically, the model group was distinctly separated from both the control and JQC-treated groups, indicating substantial microbiota disruption induced by cirrhosis (Figures 7A,B). In contrast, JQC intervention shifted the microbial community structure toward that of the control group, suggesting its potential to prevent gut dysbiosis and restore microbial homeostasis in cirrhotic mice.

The Venn diagram illustrating operational taxonomic unit (OTU) distribution revealed 194 shared OTUs across the control, model, and JQC groups. Unique OTUs numbered 461 in the control group, 700 in the model group, and 652 in the JQC group (Figure 7C). These results indicate that cirrhosis led to an increase in OTU number and microbial diversity compared to the control. Treatment with JQC partially reversed this change, reducing OTU count and modulating gut microbiota composition in cirrhotic mice.

Figures 7D–F illustrate the structural changes in the gut microbiota following JQC intervention at the phylum, family, and genus levels. At the phylum level, Bacillota, Bacteroidota, and Campylobacterota predominated across all groups. The model group showed increased relative abundance of Bacillota, Campylobacterota, and Patescibacteria, along with decreased Bacteroidota, Pseudomonadota, and Verrucomicrobiota compared to the control. These alterations were reversed by JQC treatment (Figure 7D). At the family level, the model group exhibited reduced abundance of Muribaculaceae, Erysipelotrichaceae, Prevotellaceae, norank_o_Clostridia_UCG-014, and Akkermansiaceae, all of which were restored after JQC administration (Figure 7E). Similarly, at the genus level, the model group displayed decreased relative abundance of norank_f_Muribaculaceae, Allobaculum, Dubosiella, norank_o_Clostridia_UCG-014, and Akkermansia, and JQC intervention significantly increased their abundance (Figure 7F). Further LEfSe analysis showed that, compared with the control group, the model group had a higher abundance of conditionally pathogenic bacteria, such as *Helicobacter* and Lachnospiraceae_NK4A136_group, which was consistent with the trend observed in the species composition analysis. JQC administration increased the abundance of beneficial bacteria such as Allobaculum, Akkermansia, and norank_f_Muribaculaceae (Figure 7G). These results indicate that JQC effectively attenuates gut microbiota dysbiosis and restores microbial ecology in cirrhotic mice.

**FIGURE 8:**
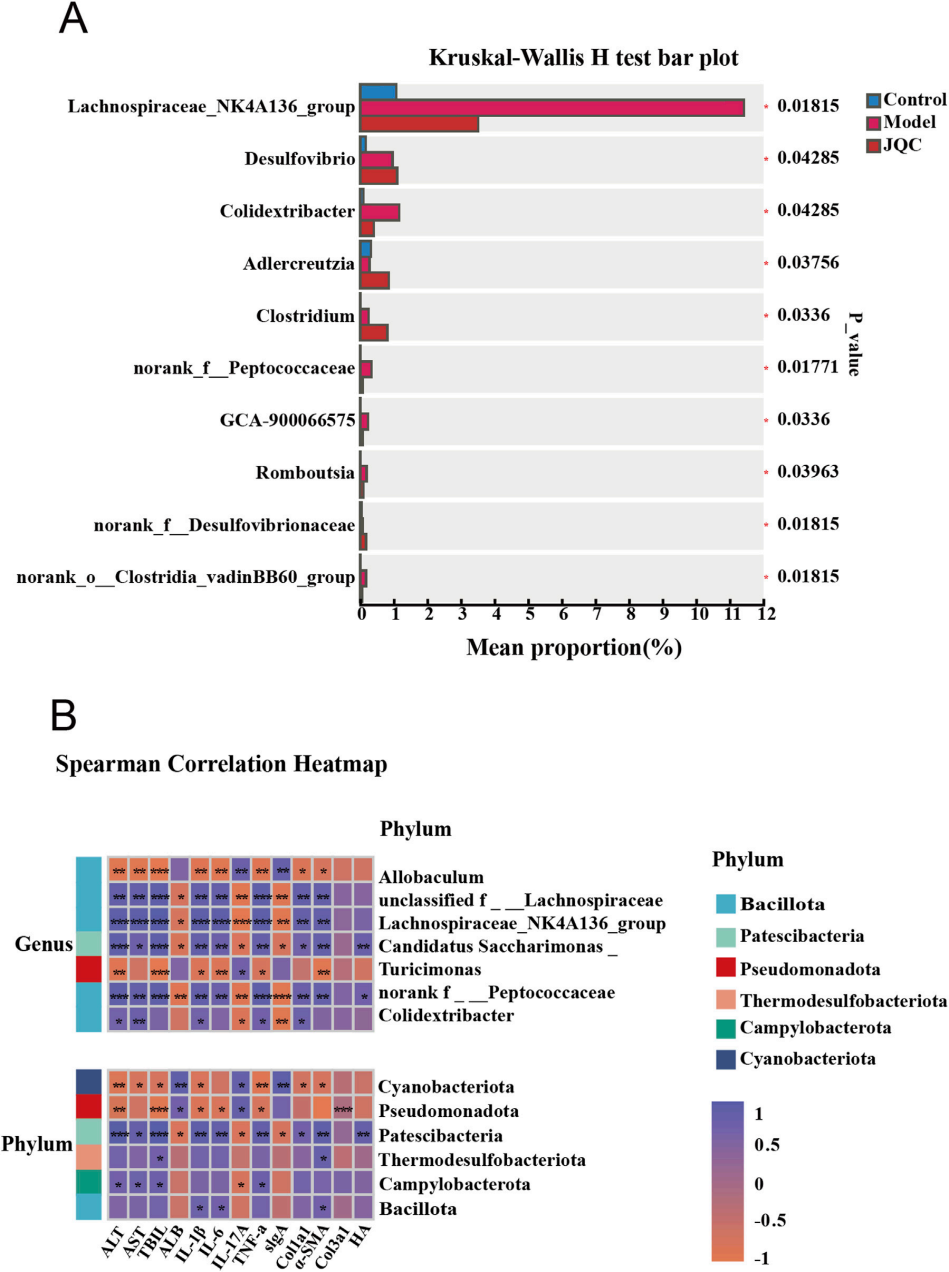
Analysis of differences in gut microbiota **(A)** and Correlation analysis of key microbial communities in each group with general indicators. **(B)** *p < 0.05, **p < 0.01, ***p < 0.001, ****p < 0.0001.

Additionally, we conducted a multivariate analysis of variance and found that the abundance of Adlercreutzia decreased in the model group compared with the control group. Meanwhile, the abundance of Lachnospiraceae_NK4A136_group, Colidextribacter, norank_f__Peptococcaceae, GCA-900066575, Romboutsia, and norank_o__Clostridia_vadinBB60_group increased. JQC treatment significantly altered gut microbiota dysbiosis in mice with CCl_4_-induced liver cirrhosis (Figure 8A). Correlation analysis indicates a close relationship between these harmful bacteria and beneficial bacteria and the progression or improvement of cirrhosis, respectively (Figure 8B).

## Discussion

Cirrhosis is a progressive, end-stage liver disease characterized by a vicious cycle of fibrosis and necroinflammation, driven by chronic liver injury. This pathological process often leads to severe complications such as ascites and hepatic encephalopathy, posing a significant threat to patient survival (Zheng et al., 2024). While preliminary studies suggest that both monomeric compounds and complex formulations derived from Chinese herbal medicine can attenuate cirrhosis progression (Wei et al., 2022; Fan et al., 2024), their precise mechanisms remain incompletely understood. JQC, a valued herb in traditional Chinese medicine, exhibits diverse pharmacological activities. However, its multi-component, multi-target nature complicates the elucidation of its therapeutic mechanisms (Zhang et al., 2025). Therefore, this study aims to investigate the anti-cirrhotic mechanisms of JQC, with a specific focus on the role of the gut-liver axis in disease progression.

We first established a classic CCl4-induced mouse model of liver cirrhosis, which closely mimics the pathological process of human liver cirrhosis. The intervention with JQC commenced in week 9 of the CCl4-induced mouse model, with continuous treatment spanning 6 weeks. The results showed that JQC treatment was effective in alleviating the progression of cirrhosis in a dose-dependent manner, as evidenced by the restoration of serum markers (ALT, AST, TBIL, HA, ALB) and the amelioration of liver histopathology, including reduced necroinflammation and fibrosis. JQC-H consistently demonstrated superior efficacy in reversing fibrotic deposition and downregulating the expression of Col1a1, Col3a1, and α-SMA, which led to its selection for subsequent investigations. Furthermore, analysis of small intestine tissue indicated that the protective mechanism is associated with suppressed inflammation and oxidative stress.

Transcriptomics provides a powerful high-throughput approach for profiling genome-wide gene expression and has become an essential tool for uncovering molecular mechanisms in complex diseases. In this study, we integrated transcriptomic sequencing with bioinformatics analysis and identified 165 key targets and three major signaling axes related to JQC treatment of cirrhosis. These core pathways included PI3K-AKT, NF-κB, and JAK-STAT signaling, all of which have been previously implicated in the pathogenesis and treatment of cirrhosis (Wei et al., 2018; Jiang et al., 2023; Zhang et al., 2023; Huang et al., 2021). Among them, the PI3K-AKT pathway, a well-established signaling cascade in liver disease progression, was also prioritized in our network pharmacology results. Thus, we selected this pathway for further experimental validation.

The PI3K/AKT axis is a highly conserved intracellular signaling pathway that senses extracellular signals to promote key physiological processes such as cell metabolism, survival, and proliferation. Research has shown that excessive activation of the PI3K/AKT signaling pathway promotes HSC proliferation, migration, and collagen synthesis (such as COL1A1 and α-SMA), resulting in ECM deposition (Shamsan et al.,2024). In addition, this pathway regulates the transcriptional activity of antioxidant enzymes (such as HO-1 and SOD2) and inflammatory factors (such as TNF-α and IL-6), thereby exerting dual effects of buffering oxidative stress and regulating inflammatory responses (Shamsan et al.,2024). The results of the transcriptomics analysis demonstrated that the PI3K-AKT pathway exhibited a marked increase in the model group compared to the control group, while the proteins associated with this pathway demonstrated a significant decrease in the JQC group compared to the model group. Consequently, we speculate that JQC may impede the progression of liver cirrhosis by inhibiting the PI3K/AKT signaling pathway. To further elucidate the potential relationship between JQC’s mechanism of action and the PI3K/AKT signaling pathway in cirrhosis, a fully automated capillary Western blotting platform was employed to assess the expression levels of key proteins within this pathway. The results demonstrated that JQC treatment led to a significant downregulation of PI3K, AKT, total protein, and their corresponding phosphorylated proteins in mice liver tissues.

It has been reported that the progression of chronic liver disease is closely related to impaired intestinal barrier function (Qi et al.,2024). LPS originates in the intestine, and its elevation is a pivotal factor in inducing liver damage, inflammation, and fibrosis. Research has demonstrated a correlation between heightened LPS levels and inflammation in extraintestinal organs, such as the liver and kidneys (An et al.,2022; Qi et al.,2024). Additionally, intestinal epithelial tight junction proteins ZO-1 and occludin are pivotal structural components that maintain intestinal barrier function and play a crucial role in intestinal barrier function (Fang et al.,2022). The present study found that CCl_4_-induced liver cirrhosis mouse models had significantly elevated LPS levels in serum, which further promoted the significant accumulation of inflammatory factors TNF-α, IL-1β, and IL-6 in the liver and downregulated the expression of tight junction proteins ZO-1 and occludin. This exacerbated liver damage and compromised the intestinal barrier and intestinal permeability. Conversely, the administration of JQC led to a reduction in LPS levels and the expression of the aforementioned inflammation-related factors, while concurrently enhancing small intestinal morphology and tight junction protein expression. In summary, the observations presented herein indicate that JQC treatment may improve the exacerbation of the intestinal mucosal barrier and intestinal permeability in murine models of cirrhosis.

Cirrhosis is closely linked to profound alterations in the gut-liver axis, in which gut microbiota dysbiosis acts as both a consequence and an accelerator of disease progression (Qi et al., 2024; Tilg et al., 2022). Evidence suggests that such dysbiosis significantly influences cirrhosis severity and clinical outcomes. It promotes the expansion of potentially pathogenic bacteria while reducing beneficial commensals, thereby increasing the risk of bacterial translocation and subsequent infections (Solé et al., 2021). What’s more, as a state of gut microbiota imbalance progresses, there is a possibility of the development of endotoxemia and metabolic toxicity accumulation, which can have deleterious effects on the liver and elevate the risk of liver failure. The present study suggests that JQC may have the capacity to regulate intestinal flora imbalance, promote the expansion of beneficial bacteria, and inhibit the excessive proliferation of harmful bacteria. This finding suggests a potential benefit for the treatment of cirrhosis.

At the same time, our research found that liver cirrhosis in mice is associated with changes in the composition of the gut microbiota, characterized by increased abundance of Bacillota and Campylobacterota, etc., and decreased abundance of Bacteroidota, norank_f__Muribaculacea, Allobaculum, and Dubosiella, etc. However, these alterations were subsequently reversed following the administration of JQC treatment. Further LEfSe analysis showed that CCl4-induced liver cirrhosis in mice resulted in significant enrichment of conditionally pathogenic bacteria in the gut, such as *Helicobacter* and Lachnospiraceae_NK4A136_group. Conversely, JQC intervention increased the abundance of beneficial bacteria such as Allobaculum, Akkermansia, and norank_f__Muribaculaceae. Correlation analysis revealed a positive correlation between the enrichment of pathogenic bacteria and the progression of cirrhosis. Conversely, a high abundance of beneficial bacteria indicated an improvement in the condition. For example, it has been reported that Patescibacteria is a pathogenic bacterium exhibiting a parasitic lifestyle dependent on other microorganisms for survival. Under conditions of microbial dysbiosis, it may proliferate excessively and negatively impact host health (Sandoz et al.,2021). In contrast, the Cyanobacteriota phylum is reported to play a key role in regulating immunity and protecting immune cells (Kar et al.,2022). Additionally, Allobaculum has been shown to enhance intestinal barrier function, which may contribute to a reduction in inflammatory responses and oxidative stress, thereby improving alcohol-induced liver damage (Chayanupatkul et al.,2022). In summary, JQC modulates the gut microbiota by enhancing microbial diversity and abundance, promoting beneficial bacteria, and suppressing harmful ones, thereby contributing to the amelioration of cirrhosis through the gut-liver axis.

Of course, our experiment still has some limitations. Firstly, The decision to forgo a positive control group in the present study was influenced by practical limitations in time and resources. Given these constraints, our priority was to allocate all available resources to comprehensively evaluate the dose-dependent effects and underlying mechanisms of JQC. We recognize the value of a clinical benchmark and intend to incorporate it in future comparative studies to further validate our findings. Secondly, the analysis of the active ingredients of JQC in this study was mainly based on mass spectrometry data reported in existing literature, which ensured the reliability of the information but lacked experimental mass spectrometry verification, constituting a significant limitation. Consequently, the research team will adopt UPLC-QTOF-MS/MS technology in the subsequent stages to establish a dedicated analysis method and enhance the detection rate. Furthermore, notwithstanding the observed correlations between JQC intervention, gut microbiota remodeling, and improved liver function, the precise regulatory network linking specific microbes to host targets and the definitive causal relationship remain to be fully elucidated. Future investigations utilizing fecal microbiota transplantation or microbiota-depletion models will be essential to establish causality and unravel the underlying microbial mechanisms.

## Conclusion

Our research indicates that JQC exerts its anti-cirrhotic effects by regulating the gut microbiota-liver axis. A mouse model of liver cirrhosis was established using a CCL_4_-induced method, and a multifaceted approach was employed to elucidate the mechanism of action of JQC in the treatment of liver cirrhosis. This approach integrated network pharmacology with transcriptomics analysis, 16S rRNA sequencing analysis, and other methods. The present study demonstrated that JQC can enhance liver function, reduce LPS levels, inflammatory responses, and oxidative stress levels, and improve small intestine morphology and tight junction protein expression. The findings of this study suggest that the intervention of JQC effectively mitigates the progression of liver cirrhosis in murine models. The study emphasizes that JQC affects the progression of liver cirrhosis through the regulation of intestinal flora, the repair of intestinal barrier function, and the inhibition of the PI3K/AKT signaling axis. This provides new strategies and value for the treatment of liver cirrhosis with JQC.

## Data Availability

The original contributions presented in the study are included in the article/Supplementary Material, further inquiries can be directed to the corresponding authors.
