# Supplementary material for: Exploring the mechanism of action of Jinqiancao granules against liver cirrhosis based on the liver-gut axis
Source: Front Pharmacol. 2025 Nov 26;16:1686535. doi: 10.3389/fphar.2025.1686535 (PMC12690457; doi:10.3389/fphar.2025.1686535)
Supplement: Supplementary file 1 [file DataSheet1.doc]

1.Primer design method

All primers used in this study (Col1a1, Col3a1, EGFR, GAPDH) were sourced from the PrimerBank database (https://pga.mgh.harvard.edu/primerbank/), a resource of experimentally validated primers. Suitable primer pairs for the mouse species were selected directly from this database.

The primer design parameters followed the default settings of the database, ensuring a primer length of 18-24 bp, a PCR product size ranging from 80 to 200 bp, and an annealing temperature between 59 and 61°C.

2.Validation of primers

To confirm primer specificity, the primer sequences obtained from PrimerBank were subjected to in silico verification using the NCBI Primer-BLAST tool (https://www.ncbi.nlm.nih.gov/tools/primer-blast/).

For the Primer-BLAST analysis, the parameters were set as follows: the organism was specified as "Mus musculus (mouse)", and the analysis was configured to ensure that each primer pair would yield a unique amplicon only for its specific target gene transcript (see accession numbers below) with no potential for non-specific binding.

3.House keeping gene selection and validation

GAPDH was selected as the reference gene based on its well-established and stable expression in mouse models, a finding confirmed by our primer validation process and consistent with its use in our group's prior research (e.g., Qi et al., Phytomedicine, 2025).

According to standard primer design principles, the primers were evaluated and found to be suitable. They demonstrated high specificity in PCR amplification, a small difference in melting temperature (Tm) between forward and reverse primers, a GC content within the optimal range of 40%–60%, and an amplicon size between 80–200 bp. Therefore, these primers were deemed appropriate for use.

Supplemental Table S1.Primer sequences

| Gene (mouse) | Primer sequence (5' to 3') | Amplicon Size (bp) | PrimerBank ID |
| --- | --- | --- | --- |
| Col1a1 | Forward：GCTCCTCTTAGGGGCCACT | 103 | 34328108a1 |
|  | Reverse：CCACGTCTCACCATTGGGG |  |  |
| Col3a1 | Forward：CTGTAACATGGAAACTGGGGAAA | 144 | 20380522a1 |
|  | Reverse：CCATAGCTGAACTGAAAACCACC |  |  |
| EGFR | Forward：GCCATCTGGGCCAAAGATACC | 101 | 10880776a1 |
|  | Reverse：GTCTTCGCATGAATAGGCCAAT |  |  |
| GAPDH | Forward：ATGGGACGATGCTGGTACTGA | 189 | 26328841a1 |
|  | Reverse：TGCTGACAACCTTGAGTGAAAT |  |  |
